# Spectrum of matrix metalloproteinase expression in primary and metastatic colon cancer: relationship to the tissuenhibitors of metalloproteinases and membrane type-1-matrix metalloproteinase

**DOI:** 10.1054/bjoc.2001.1831

**Published:** 2001-06

**Authors:** H M Collins, T M Morris, S A Watson

**Affiliations:** Division of GI Surgery, The Academic Unit of Cancer Studies, University Hospital, Nottingham, NG7 2UH, UK

**Keywords:** matrix metalloproteinases, stromal fibroblasts, cancer cell invasion metastasis, quantitative RT-PCR

## Abstract

The matrix metalloproteinases, MMP-2 and MMP-9, are capable of degrading components of the basement membrane, a vital barrier breached during the progression of colorectal cancer. The regulation of MMP-2 activation and subsequent targets is vital to understanding the metastatic process. MMP-2 was not expressed by colorectal cancer cells (C170 and C170HM _2_) in vitro but by stromal fibroblasts (46BR.1GI). There was induction of this MMP upon transwell co-cultivation of the colon cancer cells with the fibroblasts but in vivo growth did not lead to a similar increase in the metastatic tumour cells (C170HM _2_), MMP-2 again being attributed to the stromal cells. MMP-2 mRNA was overexpressed in human colorectal tumours compared to normal colorectal tissue, which correlated with Dukes' stage and immunolocalized to the stromal compartment of the tumour tissue. The active form of the MMP-2 enzyme was also present in the colorectal tumour tissue (7/8) but essentially absent in all normal colon samples examined (1/8). MMP-2 activation was not related to an increase in MT-1-MMP mRNA or a decrease in the specific inhibitor TIMP-2 in human tissue. There was however an increase in MMP-2/TIMP-2 ratio in tumour compared to normal. MMP-9, a target of active MMP-2, was present in the metastatic cell line but expression was down-regulated in the tumour cells in vivo, gelatin analysis revealed that MMP-9 was almost entirely attributable to the murine host, confirmed by PCR. There was no increase in mRNA for MMP-9 or its specific inhibitor TIMP-1 in colorectal tumour tissue compared to normal, MMP-9 protein localized to the inflammatory infiltrate. Fibroblast cells may provide malignant epithelial cells with a ready source of enzyme which is crucial to the metastatic process. © 2001 Cancer Research Campaign http://www.bjcancer.com


*British Journal of Cancer* (2001) **84**(12), 1664–1670
© 2001 Cancer Research Campaign
doi: 10.1054/ bjoc.2001.1831, available online at http://www.idealibrary.com on IDEAL®

http://www.bjcancer.com

### Spectrum of matrix metalloproteinase expression in primary and metastatic colon cancer: relationship to the tissue inhibitors of metalloproteinases and membrane type-1-matrix metalloproteinase

**HM Collins, TM Morris and SA Watson**

The Academic Unit of Cancer Studies, Division of GI Surgery, University Hospital, Nottingham, NG7 2UH, UK

**Summary** The matrix metalloproteinases, MMP-2 and MMP-9, are capable of degrading components of the basement membrane, a vital barrier breached during the progression of colorectal cancer. The regulation of MMP-2 activation and subsequent targets is vital to understanding the metastatic process. MMP-2 was not expressed by colorectal cancer cells (C170 and $C170HM_2$) in vitro but by stromal fibroblasts (46BR.1GI). There was induction of this MMP upon transwell co-cultivation of the colon cancer cells with the fibroblasts but in vivo growth did not lead to a similar increase in the metastatic tumour cells ($C170HM_2$), MMP-2 again being attributed to the stromal cells. MMP-2 mRNA was overexpressed in human colorectal tumours compared to normal colorectal tissue, which correlated with Dukes' stage and immunolocalized to the stromal compartment of the tumour tissue. The active form of the MMP-2 enzyme was also present in the colorectal tumour tissue (7/8) but essentially absent in all normal colon samples examined (1/8). MMP-2 activation was not related to an increase in MT-1-MMP mRNA or a decrease in the specific inhibitor TIMP-2 in human tissue. There was however an increase in MMP-2 / TIMP-2 ratio in tumour compared to normal. MMP-9, a target of active MMP-2, was present in the metastatic cell line but expression was down-regulated in the tumour cells in vivo, gelatin analysis revealed that MMP-9 was almost entirely attributable to the murine host, confirmed by PCR. There was no increase in mRNA for MMP-9 or its specific inhibitor TIMP-1 in colorectal tumour tissue compared to normal, MMP-9 protein localized to the inflammatory infiltrate. Fibroblast cells may provide malignant epithelial cells with a ready source of enzyme which is crucial to the metastatic process. © 2001 Cancer Research Campaign http://www.bjcancer.com

**Keywords**: matrix metalloproteinases; stromal fibroblasts; cancer cell invasion metastasis; quantitative RT-PCR

Matrix metalloproteinases (MMPs) are a family of proteolytic enzymes which are associated with tissue remodelling in both normal and pathological processes (Harris, 1990; Page, 1991). These enzymes, which degrade the extracellular matrix, are thought to play an important role in conferring metastatic potential to tumour cells and their overexpression has been correlated with the stage of progression in many tumour types (Hewitt et al, 1991; Davies et al, 1993; Stetler-Stevenson et al, 1993). Colorectal carcinomas comprise malignant epithelial cells and stroma and it is the stromal component which may prove critical in the spread of malignant cells to distant sites. These stromal cells express matrix degrading enzymes more frequently than cancer cells (Heppner et al, 1996; Ornstein et al, 1999; Saito et al, 2000) and it has been found that tumour derived diffusible factors such as EGF, TGF-$\alpha$, TNF-$\alpha$, PDGF and IL-1$\beta$ influence this expression (reviewed by Westermarck and Kahari, 1999).

Among the extensive MMP family (23 to date) MMP-2 is proving to be important in the degradative process and is predominantly expressed by the stromal component of tumours. Type IV collagen, which is the specific substrate of MMP-2, is a major constituent of the basement membrane, which is breached during invasive cancer. The active enzyme seems to have the tightest correlation with the malignant phenotype (Liabakk et al, 1996; Parsons et al, 1998) and may initiate an activation cascade which is known to exist within this enzyme family. Therefore, the control of MMP-2, along with its interaction with its specific activator MT-1-MMP and its specific inhibitor TIMP-2 may prove crucial in understanding the degradative process. TIMP-2 specifically inhibits MMP-2 in a 1:1 ratio but also contributes to its activation when present at low levels.

The second gelatinase (MMP-9) is differentially expressed by the tumour, it is expressed predominantly by inflammatory cells but can be induced by autocrine (Shimizu et al, 1996) and paracrine (Lengyel et al, 1995) stimulation. It is known to be a target of the active MMP-2 (Fridman et al, 1995) but is also susceptible to activation by other proteinases (reviewed by Birkedal-Hansen et al, 1993).

To further understand the contribution of tumour and stromal cells to the invasive process we analysed MMPs, their inhibitors and activators in systems of increasing complexity. MMP expression was examined in 2 colorectal cancer cell lines, and it was determined whether this profile was changed under the influence of stromal cells both in vitro and in vivo. The competitive-based RT-PCR assay described by Wells et al (1996) was the technique used to quantify the MMPs relative to the housekeeping gene GAPDH. This technique relies on a competition reaction in which a synthetic standard cDNA is co-amplified with the target cDNA in the same PCR reaction. The standard multi-competitor cDNA contains priming sites for human matrix metalloproteinases and

*Received 28 September 2000*
*Revised 5 March 2001*
*Accepted 7 March 2001*

*Correspondence to:* HM Collins

GAPDH. MMP and TIMP expression was also measured in both normal and tumour colorectal tissue.

### MATERIALS AND METHODS

### Cells and culture conditions

2 colorectal cancer cell lines were utilized in this study; C170 was established from a poorly differentiated human colorectal primary tumour (Durrant et al, 1986). $C170HM_2$ is a metastatic derivative of C170 which was established by selecting liver-invading tumours (Watson et al, 1993). The human fibroblast cell line 46BR.1GI (ECACC, UK) was used as an example of a stromal cell type. Cells were cultured in RPMI 1640 medium (Sigma, Poole, Dorset) containing 10% heat inactivated fetal calf serum (Sigma), at 37˚C in a humidified atmosphere of 5% $CO_2$. Cells to be grown as subcutaneous tumours were injected at a concentration of $2 \times 10^6$ in 200 µl 0.5% NaCl into the flank of MF1 nude mice, bred within the Cancer Studies Unit, University of Nottingham. After 15–20 days growth the mice were sacrificed and the resultant tumour was dissected free and fixed as described below. To initiate liver-invasive growth, $C170HM_2$ cells were injected into the peritoneal cavity ($10^6$ cells in 1 ml 0.5% NaCl), as previously described by Watson et al (1993), after 40 days the mice were sacrificed and liver-invasive tumours dissected free from the normal liver parenchyma. Following excision and dissection, tumours were snap frozen for mRNA extraction or cork mounted and snap frozen in isopentane pre-cooled in liquid nitrogen, for SDS PAGE gelatin zymography and immunohistochemistry (described below). All animal work was carried out in accordance with UKCCCR guidelines.

### Co-cultivation assay

46BR.1GI was co-cultivated in serum free medium (1:1 HAMS F12:RPMI 1640) with the colorectal cancer cell lines C170 and $C170HM_2$ in transwell plates (Costar, UK) separated by 3 µm porous filters. All assays were conducted for 72 hours, at which point each cell type was harvested and mRNA extracted. In addition to transwell co-cultivation a system where colorectal cells and fibroblasts were in cell–cell contact was set up. Conditioned media from each assay was subjected to gelatin zymography.

### Clinical samples

Fresh tissue samples were collected from patients undergoing routine resection for colorectal cancer in the Section of Surgery at the Queens Medical Centre, Nottingham. All samples underwent routine histological examination and the tumours were classified independently by a pathologist according to Dukes' stage. There were 4 Dukes' stage B and 4 Dukes' stage C tumours. Control normal colorectal tissue ($n = 8$) was obtained from sites remote from the tumour.

### mRNA extraction

For preparation of mRNA the tissue samples were finely ground in liquid nitrogen using a pre-cooled pestle and mortar. The tissue was then transferred to extraction buffer and homogenized for 30 seconds. The mRNA was extracted from cell line pellets and tissue samples using guanidium thiocyanate and purified using oligo(dT)-cellulose chromatography (Amersham Pharmacia, UK). Double-stranded cDNA was synthesized from mRNA using the Riboclone® cDNA synthesis system MMLV ($H^-$) (Promega, UK).

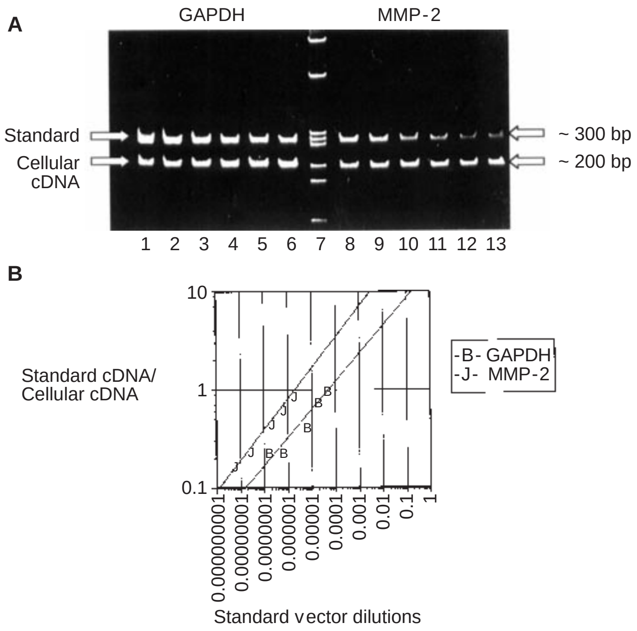

**Figure 1** **(A)** Representative example of quantitative RT-PCR using primers specific to GAPDH and MMP-2. The PCR products were separated on a polyacrylamide gel stained with ethidium bromide and visualized under u.v. light. PCR reactions from lanes 1–6 and 8–13 contain a constant amount of cellular cDNA (bottom row) and a 3-fold dilution series of standard competitor cDNA (top row) for each primer. Lane 7 – molecular weight marker. **(B)** The corresponding log-log plot used for quantitation

### RT-PCR

The double-stranded cellular cDNA was subjected to 35 cycles of PCR amplification in a Hybaid thermal cycler. Each cycle consisted of 30 s denaturation at 95˚C, 30 s annealing at 57˚C and 2 min of primer extension at 72˚C. Each 50 µl reaction contained 3.5 mM $MgCl_2$, 50 mM Tris HCl, pH 9.1, 200 µM each dNTP, 50 pmol each primer and 1 unit Taq DNA polymerase (Helena Biosciences, UK). An initial RT-PCR screen was performed with a wide range of MMPs (MMP -1, -2, -7, -9, MT-1-MMP, and -2) and TIMPs -1 and -2. All human primers were designed and kindly donated by British Biotech Ltd (Oxford, UK). The mRNA levels for MMP-2, MMP-9, MT-1-MMP, TIMP-1 and TIMP-2 were then measured by quantitative PCR as described by Wells et al (1996). MMP-9 was also examined from in vivo tumours using the murine specific primers 5′-GGGCTTAGAT-CATTCCAGCG-3′ and 5′-CCGTGGGAGGTATAGTGGGA-3′.

### Quantitative RT-PCR

Quantitation was achieved using a standard multi-competitor cDNA construct (PQH1 or PQH6) kindly donated by British Biotech Ltd (Oxford, UK). 3-fold serial dilutions of the standard competitor starting at 200 pg were combined with a constant amount of cellular cDNA in 50 µl reactions. These reactions contained the same constituents as outlined above for RT-PCR plus 1 $\mu Ci^{32}P$ $\alpha dCTP$ 3000 Ci $mmol^{-1}$ (Amersham, UK) for accurate quantitation. The PCR products were separated on precast 6%

© 2001 Cancer Research Campaign 

acrylamide gels (Novex, Germany) and visualized using ethidium bromide (Figure 1A). The fluorescent bands were excised, added to 400 µl Microscint 40 (Packard, UK) and counts per minute (cpm) determined using a Packard Top Count. The counts were normalized for relative dCTP concentration of the standard and cellular cDNAs. The dilution of standard was plotted on a log-log scale against the ratio of standard to cellular cDNA (Figure 1B). The value of the dilution achieved when the ratio of standard to cellular cDNA is equal to 1 was calculated. The MMPs and TIMPs were then expressed as a fraction of GAPDH (Glyceraldehyde-3-phosphate dehydrogenase) in each sample.

### Statistical analysis

The significance of median gene expression levels between normal and Dukes' stage B and C were statistically analysed using the Mann–Whitney, 2 sample, non-parametric test. This test was also used to test relationships between the MMPs and their specific inhibitors.

### Gelatin zymography

Gelatinolytic activity of secreted proteinases was analysed by zymography on gels co-polymerized with 0.1% gelatin as described previously by Brown et al (1990). Conditioned media from the cells grown in vitro were mixed with equal amounts of SDS sample buffer (10% (w/v) SDS, 0.5M Tris-HCL (pH 6.8), 10% (v/v) glycerol and 1% Bromophenol blue) (Novex). 10 µm cryostat tissue sections were mixed with 100 µl SDS buffer. The tissue samples were allowed to stand for 10 minutes in solution, aspirated through a fine needle and centrifuged for 1 minute at 13 000 rpm. 25 µl of sample was loaded onto the gel and electrophoresed. The gelatinases that were separated on the gel were renatured for 30 min in 2.5% Triton X-100 at room temperature. This was followed by a 30 min equilibration in a developing buffer containing 50 mM Tris (pH 7.6), 0.2 M NaCl and 5 mM $CaCl_2$. The gel was incubated for a further 18 hours in a fresh solution of developing buffer. Gels were stained with Coomassie Brilliant Blue. Human Recombinant MMP-2 (72 kDa) kindly donated by British Biotech Ltd (Oxford, UK) was used as a molecular weight marker.

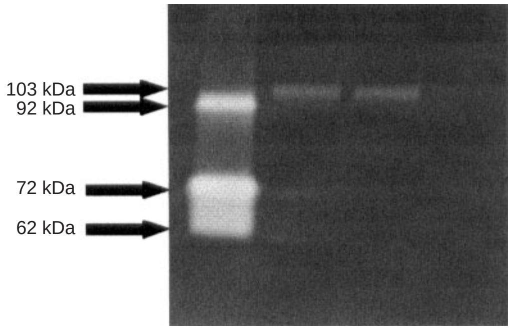

**Figure 2** Lane 1 Gelatinase standard displaying the pro form of MMP-9 (92 kDa) and both the pro and active forms of MMP-2 (72 kDa and 62 kDa). Lanes 2 and 3 $C170HM_2$ in vivo, Lane 4 $C170HM_2$ in vitro

### Immunohistochemistry

Frozen sections (4 µm) were cut from the same blocks used for zymography. Routine histology confirmed the presence of normal and tumour tissue in the respective specimens. Immunocytochemistry was performed on all tumour and normal sections for MMP-2 and MMP-9 using mouse monoclonal antibodies from R&D systems, (Abingdon, UK). The antibodies specifically bind to both the pro and active forms of each enzyme. Briefly sections were air dried and fixed in acetone for 10 minutes. Antibodies were used at 2 µg $ml^{-1}$ concentration in 10 mM PBS. The sections were labelled with the Avidin/Biotin complex (ABC) (Dakopatts, UK) and detected using the DAB detection system (Sigma, UK). Rabbit serum was used as a negative control and the specificity of the staining was confirmed by blocking with the respective antigen.

### RESULTS

### MMP mRNA in vitro

The 2 colorectal cancer cell lines C170 and $C170HM_2$ were examined for a range of MMPs when grown in vitro and in vivo. Table 1

**Table 1** Non-competitive RT-PCR screen for MMPs, MT-MMPs and TIMPs in cell lines grown in vitro

| Cell line | MMP-1 | MMP-2 | MMP-7 | MMP-9 | MT-1-MMP | MT-2-MMP | TIMP-1 | TIMP-2 |
|---|---|---|---|---|---|---|---|---|
| 46BR.IGI | + | + | + | + | – | – | + | + |
| C170 | – | – | + | – | + | + | + | + |
| $C170HM_2$ | + | – | + | + | + | + | + | + |

**Table 2** Quantitative analysis of MMP-2 and MMP-9 mRNA from colorectal cancer cell lines grown in vitro and in vivo

| | C170 (in vitro) | C170 (in vivo) Subcutaneous | $C170HM_2$ (in vitro) | $C170HM_2$ (in vivo) Subcutaneous | $C170HM_2$ (in vivo) Liver invasive |
|---|---|---|---|---|---|
| MMP-2 | ND | $2.76 \times 10^{-2}$ | ND | ND | ND |
| MMP-9 | ND | $2.59 \times 10^{-6}$ | $2.0 \times 10^{-2}$ | $1.37 \times 10^{-6}$ | $7.13 \times 10^{-7}$ |

 © 2001 Cancer Research Campaign shows the range of MMPs expressed at the gene level in C170 compared to its metastatic derivative $C170HM_2$. MMP-1 and MMP-9 were present in the metastatic cell line but absent in the non-invasive cell line C170. MMP-2 was not detected at the gene level by in vitro cell monocultures of the colorectal cancer cell lines. There was expression of almost all the MMPs, including MMP-2, at the gene level by the fibroblast cell line, MT-1-MMP and MT-2-MMP were notably absent.

### MMP mRNA in vivo

Comparisons between the in vitro and the in vivo situation of the non-invasive cell line C170 revealed low levels of MMPs-1, –2 and –9 in the subcutaneous graft. The main difference between C170 and $C170HM_2$ was the expression of MMP-2 (albeit low – $2.76 \times 10^{-6}$) in the C170 xenograft, this MMP was not evident from either the primary or the secondary tumours of $C170HM_2$ (see Table 2).

A quantitative analysis of MMP mRNA from the $C170HM_2$ tumours grown at primary (subcutaneous) and secondary sites (liver) revealed that the same range of MMPs were expressed as $C170HM_2$ grown in vitro. Levels of $C170HM_2$ MMP-9 mRNA were however lower in vivo, subcutaneous ($1.37 \times 10^{-6}$) and liver ($7.13 \times 10^{-7}$), than in vitro ($2 \times 10^{-2}$) (Table 2). Therefore the expression of this MMP from the murine components of the tumour were examined to account for any stromal influence on expression. RT-PCR using mouse-specific primers confirmed MMP-9 in the mouse stromal component of the xenograft tissue at both the primary and secondary sites; the liver tissue was negative. To confirm no cross-reaction with human MMP-9 expression, the human cell lines grown in vitro were also examined. There was no expression found in any of the cell lines (results not shown).

### Gelatinase expression in vitro and in vivo

The gelatinases were examined from cell line supernatants and xenograft tissue using gelatin zymography. Both gelatinase proforms were found in the fibroblast supernatant. These enzymes were not evident in colorectal cancer cell line supernatants, however the pro-forms of MMP-9 (murine) and MMP-2 along with active MMP-2 (62 kDa) were present in vivo (Figure 2).

### MMP expression following transwell co-cultivation

In the transwell co-cultivation system there was de novo expression of MMP-2 by the colorectal cancer cell lines at the gene and protein level. Both cell lines also expressed the proform of MMP-9 in the co-cultivation system (Table 3). Monocultures of the fibroblast cell line expressed mRNA for MMP-2 and –9 but did not express MT-1-MMP mRNA, however co-culture with both colorectal cancer lines induced de novo expression of MT-1-MMP while retaining expression of MMP-2 and –9. Expression of MT-1-MMP by the fibroblast cell line did not activate fibroblast or tumour-derived pro-MMP-2. Colorectal cancer cells co-cultured in cell–cell contact with fibroblasts expressed non-activated MMP-2 in vitro.

### Non-competitive screen in normal versus tumour

To look at MMP expression with a fully functional extracellular matrix, paired tumour and uninvolved mucosa, comprising mostly of Dukes'B and Dukes'C, moderately differentiated colorectal adenocarcinomas were analysed, by RT-PCR for the expression of a wide range of MMPs, their activators and inhibitors. The initial non-competitive screen revealed that mRNA was present for all the MMPs and their inhibitors in all of the tumour samples. However, expression of MMP mRNA was more varied in the normal samples with a more limited expression pattern (Figure 3): MMP-1 (37.5%), MMP-2 (50%), MMP-9 (100%), and MMP-7 (100%). MT-1-MMP was expressed in all normal and tumour samples along with TIMP-1 and TIMP-2, MT-2-MMP was present

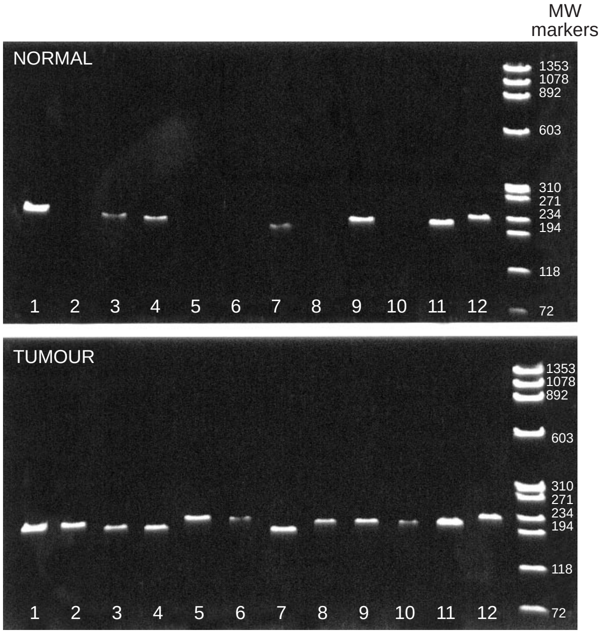

**Figure 3** Non-competitive RT-PCR screen of both normal and tumour colorectal tissue. Lane 1 GAPDH, Lane 2 MMP-1, Lane 3 MMP-2, Lane 4 MMP-9, Lanes 5–7 MMP-3, -10, -11, Lane 8 MMP-7, Lane 9–10 MT-MMP-1, -2, Lanes 11–12 TIMP-1, -2 MW marker ØX174 DNA Hae III Digest

**Table 3** Cocultivation results

| | 46BRIGI with C170 | 46BRIGI with $C170HM_2$ | C170 with 46BRIGI | $C170HM_2$ with 46BRIGI |
|---|---|---|---|---|
| MMP-2 | + | + | + | + |
| MMP-9 | + | + | + | + |
| MT-1-MMP | + | + | + | + |

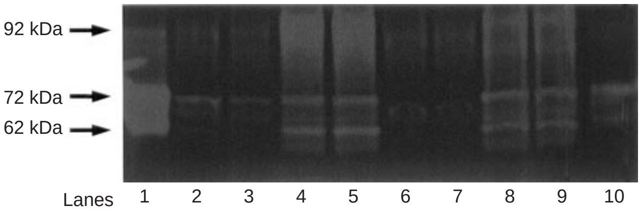

**Figure 4** Lanes 1 and 10 are standard lanes displaying 92 kDa (MMP-9) and both the pro and active forms of MMP-2 (72 kDa and 62 kDa), Lanes 2–3 and 6–7 are normal colorectal tissue samples while lanes 4–5 and lanes 8–9 are colorectal tumour samples. The tumour samples typically display active MMP-2 which is absent in normal samples

© 2001 Cancer Research Campaign in all but one normal sample and all tumour tissue. The amplification of GAPDH in all sample tissue confirmed the presence of intact mRNA in each sample.

### Over-expression of MMP-2 mRNA

A competitive RT-PCR assay for MMP-2, MMP-9, MT-1-MMP, TIMP-1 and TIMP-2 positive in the non-competitive screen, was set up to measure the levels of mRNA present related to the house-keeping gene *GAPDH*. The median level of MMP-2 in tumour tissue (Dukes'B and C) was significantly higher than the median level in normal tissue ($P = 0.0036$). The median level in samples from Dukes' stage C was significantly higher than Dukes' stage B ($P = 0.0304$) and normal samples ($P = 0.0265$). The difference between Dukes' stage B and normal samples failed to reach significance. MT-1-MMP, the specific activator of MMP-2, failed to reach significance at any stage when the differences in median levels in tumour were compared to normal tissue. TIMP-2 was expressed in both normal and tumour tissue, and there was no statistical difference between the medians of the 2 groups. However, statistical significance was achieved when the median levels of the ratio between TIMP-2/MMP-2 in normal tissue was compared to the ratio of that seen in colorectal tumour tissue ($P = 0.0072$).

The median level of MMP-9 in tumour compared to normal failed to reach significance at any stage. The mRNA levels of TIMP-1 were generally a lot higher than those found for any of the MMPs (median levels, normal = 0.265, tumour = 0.347), however there was no statistical significance in levels found between the normal and the tumour tissue. The ratio between the inhibitor and MMP-9 also failed to reach significance.

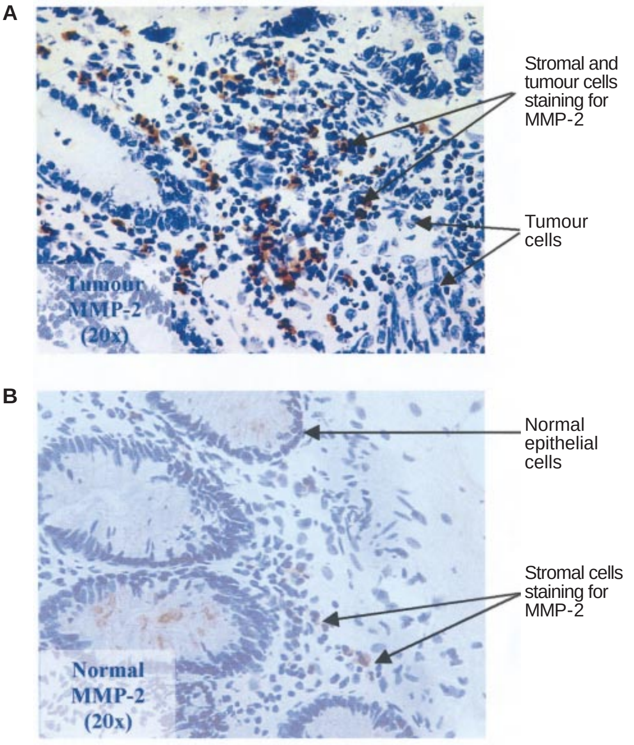

**Figure 5 (A–B)**. Immunocytochemistry with anti-MMP-2 antibody. **(A)** Colorectal tumour samples displaying immunoreactivity for MMP-2, predominantly localized to stromal cells. **(B)** Normal colonic mucosa showing only occasional positivity in stromal cells. Immunoreactivity is stained as brown – DAB

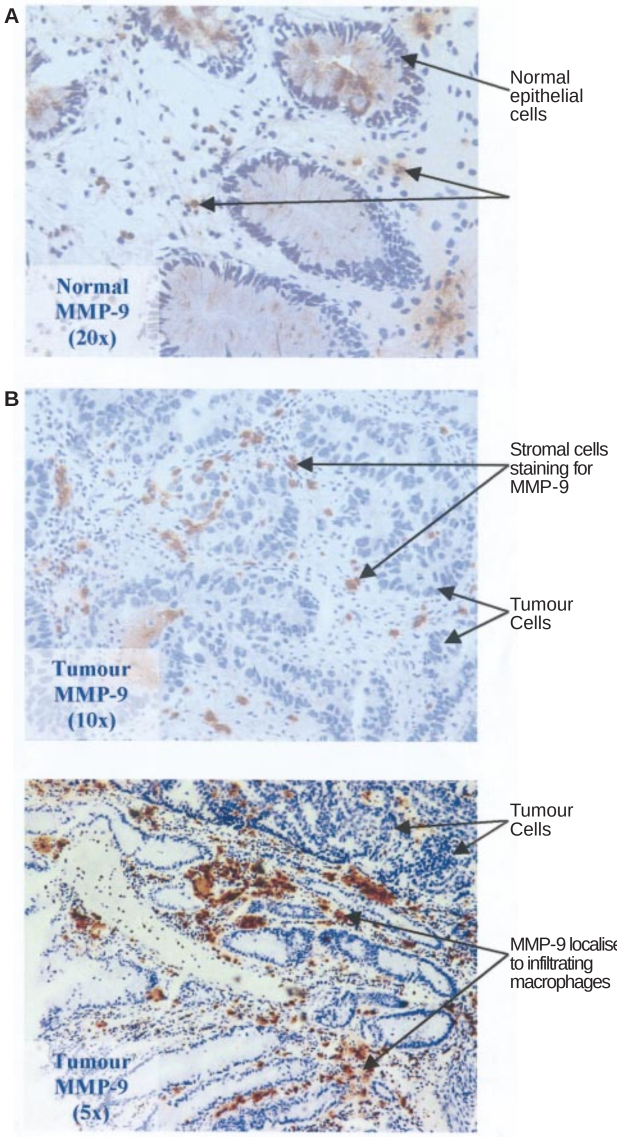

**Figure 6 (A–C)**. Immunocytochemistry with anti-MMP-9 antibody. **(A)** Normal colonic mucosa showing only occasional positivity in stromal cells. **(B–C)** Colorectal tumour samples displaying immunoreactivity for MMP-9, predominantly localized to infiltrating macrophages. Immunoreactivity is stained as brown – DAB

### Gelatinase expression in human colorectal tissue

The colorectal tissue analysed by quantitative RT-PCR was also examined by zymography. In our examination of MMP-2 protein we found that 7/8 tumours expressed the active form of MMP-2 compared to only one normal sample. This confirmed previous findings by this group (Parsons et al, 1998) that the active form of MMP-2 had the tightest correlation with the malignant phenotype (see Figure 4).

### Immunohistochemistry

MMP-2 and MMP-9 were analysed at the protein level in colorectal resection margin normal mucosa and tumour tissue by immunohistochemistry using monoclonal antibodies and the DAB detection system. In this study the MMP-2 protein localized to both the stromal and the epithelial components of tumour tissue

 © 2001 Cancer Research Campaign

(Figure 5A) with only occasional positivity in the normal samples (Figure 5B). The histology performed with MMP-9 clearly identifies the cellular source as inflammatory cells in both the normal and the tumour tissue (Figure 6 A–C).

### DISCUSSION

MMP expression has been linked to the progression of colorectal cancer but the interactions between MMPs from tumour and host cells and the processes which activate these enzymes still remains unclear. Previous research by our group showed significant over-expression of pro-MMP-9 and pro and active MMP-2 in colorectal tumours compared to normal mucosa (Parsons et al, 1998). The present study aimed to establish the range and relative expression of MMPs produced by stromal cells and their influence on MMPs produced by colorectal cancer cells at the gene level, both in vitro (co-cultivation system) and in vivo (subcutaneous xenografts), and to determine the effect tumour/stromal interactions have on gelatinase activation.

MMP-2 mRNA was expressed by fibroblasts, but not colorectal cancer lines in vitro, this corresponds with previous studies where expression was predominantly stromal (Heppner et al, 1996; Ornstein et al, 1999). The colorectal cancer lines did express mRNA for the MMP-2 activator, MT-1-MMP, which was absent from the fibroblast monoculture. De novo expression of fibroblast MT-1-MMP in transwell co-cultivations indicates that this expression may be under the control of tumour-derived factors. Polette et al (1997) showed a similar induction in fibroblasts co-cultured with breast adenocarcinoma cells and several other studies have associated increases in MMP-2 expression and activation with tumour-derived factors (Noel et al, 1994; Biswas et al, 1995). However, it is the active form of MT-1-MMP and not the gene expression which is the key to MMP-2 activation. Lehti et al (1998) demonstrated that fibroblast MT-1-MMP was synthesized at a reduced rate compared to fibrosarcomas and is immediately complexed with membrane associated TIMP-2 and MMP-2, preventing activation, suggesting different post-translational regulation.

Although fibroblast-derived soluble factors induced the expression of pro-MMP-2 in tumour cells there was no evidence of activation when cultured in contact with fibroblasts, activation may require additional ECM components as the pro and active forms of MMP-2 were present in vivo. MMP-2 expression may be host in origin or up-regulated in tumour cells due to host factors. McDonnell et al (1999) found that although not expressed in vitro, MMP-2 was present in subcutaneous xenografts with activation evident in caecal tumours, MMP-2 mRNA in vivo was not examined. The present study however, revealed that human MMP-2 mRNA was expressed de novo in vivo by a human colorectal cancer cell line. The MMP-2 protein evident in vivo is likely to be expressed by both the mouse stroma and human epithelial tissue. Activation of host MMP-2 may be due to the correct combination of tumour-derived MT-1-MMP and TIMP-2 along with components of the ECM as suggested by the in vitro studies. MMP-2 activation is promoted by culturing cells on fibronection (Stanton et al, 1998), reinforcing the view that activation may be regulated by cell–matrix interactions, with the aggregation of β1 integrins implicated as a key event (Ellerbroek et al, 1999).

In correlation with earlier findings of over-expression of MMP-2 protein in colorectal cancer (Parsons et al, 1998), MMP-2 mRNA was significantly increased in Dukes' C tumours compared to normal colonic mucosa. MMP-2 was also significantly increased in Dukes' C compared to Dukes'B with MMP-2 protein localized to the stromal component of these tumours. MMP-2 has been related to tumour progression (D'Errico et al, 1991) with in situ hybridization localizing expression to the tissue stroma adjacent to the invasive front (Poulsom et al, 1992). Increased gelatinase expression has also been confirmed in the stromal components of tumours (Pyke et al, 1993; Zeng and Guillem, 1995). MMP-2 activation was found in 7/8 tumours in the present study compared to only one normal sample. This increase in MMP-2 activation in tumours may be due to the significant increase ($P$ = 0.0072) in MMP-2/TIMP-2 ratio allowing free MMP-2 to bind to the MT-1-MMP complex in tumour tissue. Other groups have investigated the significance of this ratio and in a recent study Masuda and Aoki (1999) found that the ratio of MMP-2/TIMP-2 was significantly higher in normal colonic tissue in patients with liver metastasis than in those without metastatic spread.

MMP-9, which is a close structural homologue to MMP-2, does not appear to be similarly expressed or activated. In contrast to MMP-2, there was no significant increase in MMP-9 mRNA in tumours compared to normal tissue. This does not correspond to protein levels discussed in the literature where overexpression correlated with disease progression (Stetler-Stevenson et al, 1993; Liabakk et al, 1996). MMP-9 is unique among MMPs in that it has a nuclear factor-κB promoter recognition site which is inducible by a range of inflammatory cytokines (Sato et al, 1994). MMP-9 may be up-regulated in adjacent normal tissue due to an infiltration of inflammatory cells responding to the tumour, MMP-9 was immunolocalized to inflammatory cells in both normal and tumour tissue. This correlates well with diseases such as inflammatory bowel disease which have reported over-expression of MMP-9 (Pender et al, 1997).

Examination of paired colorectal cancer lines revealed that the metastatic line expressed MMP-1 and MMP-9 mRNA which were absent in the parental line in vitro. The presence of MMP-9 mRNA in the metastatic cells may be due to autocrine stimulation by growth factors or cytokines known to induce MMPs (Birkedal-Hansen et al, 1993). Hyuga et al (1994) found that an autocrine factor enhanced the expression and secretion of MMP-9 in serum-free media conditioned with murine metastatic colon carcinoma cells which was not repeated with cells of low metastatic potential. Further work by the same group linked this enhancement to TGFβ1 (Shimizu et al, 1996). We have demonstrated paracrine stimulation of MMP-9 in co-cultivation studies with an increase in protein from $C170HM_2$ cells, along with de novo mRNA and protein expression from C170 cells. Lengyel et al (1995) has shown that paracrine factors secreted by fibroblasts induced MMP-9 in a squamous cell carcinoma line. This paracrine stimulation in vitro was also reflected in vivo. The liver metastatic tumours grown in vivo expressed pro-MMP-9 which was expected as this line expressed human MMP-9 mRNA in vitro, however, MMP-9 was now also expressed by C170 tumours. Examination of xenograft tissue using mouse-specific primers revealed that the host tissue was also responsible for producing MMP-9. When MMP-9 gene expression was quantified in the $C170HM_2$ cell line both in vitro and in vivo there was a notable decrease in human MMP-9 in the xenograft. Analysis of in vivo tumours via zymography revealed that pro-MMP-9 was ~103 kDa known to be the murine form (Masure et al, 1993), thus MMP-9 protein is of mouse stromal origin, despite the epithelial cells expressing the

© 2001 Cancer Research Campaign 

gene. McDonnell et al (1999) found that MMP-9 in subcutaneous and caecal tumours was host derived, confirmed by RT-PCR and in situ hybridization, where the signal was stromal and found predominantly at the tumour/stromal interface. In a study by Segain et al (1996), it was demonstrated that fibroblast MMP-9 was induced by direct cell–cell contact with cell lines derived from primary colorectal tumours and that cell surface molecules may trigger this signalling pathway.

In conclusion we have demonstrated that MMP expression is influenced by autocrine and paracrine stimulation and that MMP-2 activation requires co-operation between epithelial and stromal cells as well as ECM components. We have illustrated the over-expression of MMP-2 mRNA in colorectal cancer compared to normal mucosa and indicated that it is the ratio of this enzyme in relation to its activator and inhibitor which is important in determining the overall degradative process. Specific inhibitors which target MMP-2 activity (or components of the ECM which facilitate this activation) may be of therapeutic benefit to patients with colorectal cancer.

### REFERENCES

Birkedal-Hanson H, Moore W, Bodden M, Windsor L, Birkedal-Hanson B, Decarlo A and Engler J (1993) Matrix metalloproteinases a review. *Crit Rev Oral Biol Med* **4**: 197–250

Biswas C, Zhang Y, DeCastro R, Guo H, Nakamura T, Kataoka H and Nabeshima K (1995) The human tumour cell-derived collagenase stimulatory factor (renamed EMMPRIN) is a member of the immunoglobulin superfamily. *Cancer Res* **55**: 434–439

Brown PD, Levy A, Margulies I, Liotta L and Stetler-Stevenson W (1990) Independent expression and cellular processing of Mr 72,000 type-IV collagenase and interstitial collagenase in human tumorigenic cell lines. *Cancer Res* **50**: 6184–6191

Davies B, Waxman J, Wasan H, Abel P, Williams G, Krausz T, Neal D, Thomas D, Hanby A and Balkwill F (1993) Levels of matrix metalloproteases in bladder cancer correlate with tumour grade and invasion. *Cancer Res* **53**: 5365–5369

D'Errico A, Garbisa S, Liotta L, Astronovo V, Stetler-Stevenson W and Grigioni W (1991) Augmentation of type IV collagenase, laminin receptor and Ki67 proliferation antigen associated with human colon, gastric and breast carcinoma progression. *Mod Pathol* **4**: 239–246

Durrant LG, Robins RA, Pimm MV, Perkins AC, Armitage NC, Hardcastle JD and Baldwin RW (1986) Antigenicity of newly established colorectal carcinoma cell lines. *Br J Cancer* **53**: 37–45

Ellerbroek SM, Fishman DA, Kearns AS, Bafetti LM and Stack MS (1999) Ovarian carcinoma regulation of Matrix metalloproteinase-2 and membrane type 1 matrix metalloproteinase through β1 integrin. *Cancer Res* **59**: 1635–1641

Fridman R, Toth M, Pena D and Mobashery S (1995) Activation of progelatinase B (MMP-9) by gelatinase A (MMP-2). *Cancer Res* **55**: 2548–2555

Harris ED Jr (1990) Rheumatoid arthritis. Pathophysiology and implications for therapy. *New Engl J Med* **322**(18): 1277–1289

Heppner KJ, Matrisian LM, Jensen RA and Rodgers WH (1996) Expression of most matrix metalloproteinase family members in breast cancer represents a tumor-induced host response. *Am J Pathol* **149**: 273–282

Hewitt R, Leach I, Powe D, Clark I, Cawston T and Turner D (1991) Distribution of collagenase and tissue inhibitor of metalloproteinases (TIMP) in colorectal tumors. *Int J Cancer* **49**: 666–672

Hyuga S, Nishikawa Y, Sakata K, Tanaka H, Yamagata S, Sugita K, Saga S, Matsuyama M and Shimizu S (1994) Autocrine factor enhancing the secretion of Mr 95,000 gelatinase (Matrix Metalloproteinase 9) in serum free medium conditioned with murine metastatic colon carcinoma cells. *Cancer Res* **54**: 3611–3616

Lehti K, Lohi J, Valtnan H and Keski-Oja J (1998) Proteolytic processing of membrane-type-1 matrix metalloproteinase is associated with gelatinase activation at the cell surface. *Biochem J* **334**: 345–353

Lengyel E, Gum R, Juarez J, Clayman G, Seiki M, Sato H, and Boyd D (1995) Induction of Mr 92,000 Type IV collagenase expression in a squamous cell carcinoma cell line by fibroblasts. *Cancer Res* **55**: 963–967

Liabakk N, Talbot I, Smith RA, Wilkinson K and Balkwill F (1996) Matrix metalloproteinases2 (MMP-2) and matrix metalloproteinase 9 (MMP-9) type IV collagenases in colorectal cancer. *Cancer Res* **56**: 190–196

Masuda H and Aoki H (1999) Host expression of Matrix metalloproteinase-2 and tissue inhibitor of metalloproteinase-2 in normal colon tissue affects metastatic potential of colorectal cancer. *Dis Colon Rectum* **42**: 393–397

Masure S, Nys G, Fiften P, Van Damme J and Opdenakker G (1993) Mouse Gelatinase B: cDNA cloning, regulation of expression and glycosylation in WEHI-3 macrophages and gene organisation. *Eur J Biochem* **218**: 129–141

McDonnell S, Chaudhry V, Mansilla-Soto J, Zeng Z-S, Shu WP and Guillem JG (1999) Metastatic and non-metastatic colorectal cancer (CRC) cells induce host metalloproteinase production in vivo. *Clin Exp Metas* **17**: 341–349

Noel A, Polette M, Lewalle J-M, Munaut C, Edmonard HP, Birembaut P and Foidart J-M (1994) Co-ordinate enhancement of gelatinase A mRNA and activity levels in human fibroblasts in response to breast-adenocarcinoma cells. *Int J Cancer* **56**: 331–336

Ornstein DL, MacNab J and Cohn KH (1999) Evidence for tumour-host co-operation in regulating MMP-2 expression in human colon cancer. *Clin Exp Metas* **17**: 202–212

Page RC (1991) The role of inflammatory mediators in the pathogenesis of periodontal disease. *J Periodont Res* **26**: 230–242

Parsons SL, Watson SA, Collins HM, Griffin NR, Clarke PA and Steele RJC (1998) Gelatinase (MMP-2 and –9) expression in gastrointestinal malignancy. *Br J Cancer* **78**(11): 1495–1502

Pender S, Tickle S, Docherty A, Howie D, Wathen N and MacDonald T (1997) A major role for matrix metalloproteinases in T-Cell injury in the gut. *J Immunol* **158**: 1582–1590

Polette M, Gilles C, Marchand V, Seiki M, Tournier JM and Birembaut P (1997) Induction of membrane-type matrix metalloproteinase 1 (MTl-MMP) expression in human fibroblasts by breast adenocarcinoma cells. *Clin Exp Metas* **15**(2): 157–163

Poulsom R, Pignatelli M, Stetler-Stevenson WG, Liotta LA, Wright PA Jeffery RE, Longcroft JM, Rodgers L and Stamp GWH (1992) Stromal expression of 72kDa Type IV collagenase (MMP-2) and TIMP-2 mRNAs in colorectal neoplasia. *Am J Pathol* **141**: 389–396

Pyke C, Ralfkiaer E, Tryggvason K and Dano K (1993) Messenger RNA for two type IV collagenases is located in stromal cells in human colon cancer. *Am J Pathol* **142**(2): 359–365

Saito K, Takeha S, Shiba K, Matsuno S, Sorsa T, Nagura H and Ohtani H (2000) Clinicopathologic significance of urokinase receptor-and MMP-9-positive stromal cells in human colorectal cancer: functional multiplicity of matrix degradation on hematogenous metastasis. *Int J Cancer* **86**(1): 24–29

Sato H, Takino T, Okada Y, Cao J, Shinagawa A, Yamamoto E and Seiki M (1994) A matrix metalloproteinase expressed on the surface of invasive tumour cells. *Nature* **370**: 61–65

Segain JP, Harb J, Gregoire M, Meflah K and Menanteau J (1996) Induction of fibroblast gelatinase B expression by direct contact with cell lines derived from primary tumour but not metastases. *Cancer Res* **56**: 5506–5512

Shimizu S, Nishikawa Y, Kuroda K, Takagi S, Kozaki K, Hyuga S, Saga S and Matsuyama M (1996) Involvement of Transforming growth factor β1 in autocrine enhancement of gelatinase B secretion by murine metastatic colon carcinoma cells. *Cancer Res* **56**: 3366–3370

Stanton H, Gavrilovic J, Atkinson SJ, d'Ortho MP, Yamada KM, Zardi L and Murphy G (1998) The activation of proMMP-2 (gelatinase A) by HT1080 cells is promoted by culture on a fibronectin substrate and is concomitant with an increase in processing of MT-1-MMP (MMP-14) to a 45kDa form. *J Cell Sci* **111**: 2789–2798

Stetler-Stevenson WG, Liotta LA and Kleiner DE (1993) Extracellular matrix 6: role of matrix metalloproteinases in tumor invasion and metastasis. *FASEB J* **7**: 1434–1441

Watson SA, Morris TM, Crosbee DM and Hardcastle JD (1993) A new hepatic invasive human colo-rectal xenograft model. *Eur J Cancer* **29**: 1740–1745

Wells GMA, Catlin G, Cossins JA, Mangan M, Ward GA, Miller KM and Clements JM (1996) Quantitation of Matrix Metalloproteinases in cultured rat astrocytes using the polymerase chain reaction with a multicompetitor cDNA standard. *Glia* **18**: 332–340

Westermarck J and Kahari VM (1999) Regulation of matrix metalloproteinase expression in tumor invasion. *FASEB J* **13**: 781–792

Zeng ZS and Guillem JG (1995) Distinct pattern of matrix metalloproteinase-9 and tissue inhibitor of metalloproteinase 1 mRNA expression in human colorectal cancer and liver metastases. *Br J Cancer* **72**: 575–582

 *© 2001 Cancer Research Campaign*
